# Recombinant Dual-target MDM2/MDMX Inhibitor Reverses Doxorubicin Resistance through Activation of the TAB1/TAK1/p38 MAPK Pathway in Wild-type p53 Multidrug-resistant Breast Cancer Cells

**DOI:** 10.7150/jca.32765

**Published:** 2020-01-01

**Authors:** Yangwei Fan, Ke Ma, Jiayu Jing, Chuying Wang, Yuan Hu, Yu Shi, Enxiao Li, Qianqian Geng

**Affiliations:** 1 Department of Medical Oncology, the First Affiliated Hospital of Xi'an Jiaotong University, Xi'an710061, China; 2 Department of Medical Oncology, the First Affiliated Hospital of Zhengzhou University, Zhengzhou450052, China; 3 Department of Nuclear Medicine, the First Affiliated Hospital of Xi'an Jiaotong University, Xi'an710061, China

**Keywords:** breast cancer, p53, MDM2, MDMX, MDR

## Abstract

Chemotherapy resistance represents a major obstacle for the treatment of patients with breast cancer (BC) and greatly restricts the therapeutic effect of the first-line chemotherapeutic agent doxorubicin (DOX). The present study aimed to investigate the feasibility of the recombinant dual-target murine double minute 2 (MDM2) and murine double minute X (MDMX) inhibitor in reversing the DOX resistance of BC. Both DOX-resistant human breast carcinoma cell lines exhibited a multidrug resistance (MDR) phenotype. The ability of the dual-target MDM2/MDMX inhibitor in reversing doxorubicin resistance was subsequently verified, (9.15 and 13.92 - fold reversal indexes) respectively. We observed that the MDM2/MDMX inhibitor in combination with DOX could suppress proliferation, promote cell cycle arrest and induce apoptosis. In addition, it was capable of reducing rhodamine123 efflux in DOX-resistance BC cell lines and further played a key role in BC nude mice model. The groups that were treated with the combination of the drugs had decreased P-glycoprotein/multidrug resistance-associated protein/cdc 2/Bcl-2 expression and increased CyclinB1/Bax expression. These effects were caused due to activation of the transforming growth factor β-activated kinase 1 (TAK1)-binding protein 1 (TAB1)/TAK1/p38 mitogen-activated protein kinase (MAPK) signaling pathway, as shown by small interfering RNA (siRNA) silencing and immumohistochemical staining of BC tissue sections. Furthermore, high MDM2/MDMX expression was positively associated with weak TAB1 expression in BC patients. Therefore, the recombinant dual-target MDM2/MDMX inhibitor could reverse doxorubicin resistance via the activation of the TAB1/TAK1/p38 MAPK pathway in wild-type p53 multidrug-resistant BC.

## Introduction

Breast cancer (BC) remains the most common malignancy among women in western countries^1^, whereas its incidence and mortality rates continue to rise rapidly in China^2^. Currently, chemotherapy is one of the predominant treatment strategies for BC. Doxorubicin (DOX) is an anthracycline antibiotic that functions by intercalating DNA^3^,^4^ and has been used as a first-line antitumor agent for the treatment of BC since its initial discovery^5^. However, the widespread application of DOX has led to drug insensitivity and/or resistance^6^, which has caused tumor recurrence and metastasis^7^. Approximately 500,000 deaths per year occur among women with BC^8^. Therefore, it is necessary to fully understand the mechanism of DOX resistance in breast tumors. The potential mechanisms associated with DOX resistance have been reported in several studies and include overexpression of ATP-binding cassette (ABC) transporter proteins, such as P-glycoprotein (P-gp) and multidrug resistance protein (MRP)^9^, dysregulation of the cell cycle^10^,^11^ and inhibition of apoptosis^12^. Although several reports have examined the occurrence of DOX resistance and relevant reversal agents in BC, their actual effects and underlying molecular mechanisms are not yet fully explored. Thus, new adjuvant regimens are required to enhance the efficacy of Dox-based chemotherapy.

It is well established that the tumor suppressor gene p53 plays a pivotal role in coordinating the cellular responses to a wide variety of stress signals^13^. However, the mutated and/or functionally inactivated p53 protein that exists in the majority of human cancers cannot suppress tumor growth, but rather accelerates tumor development^14^. The restoration of wild-type p53 tumor suppressor function has therefore emerged as an attractive anticancer strategy^15^. The p53 protein is mainly downregulated by MDM2 and MDMX in a complex network^16^,^17^. Numerous studies have focused on the pharmacological inhibition of the p53-MDM2 interaction in order to reactivate p53 expression in human wild-type p53 tumors^18^. However, only a few of these have been successful^19^,^20^. In the present study, we hypothesized that optimal p53 reactivation may only be achieved by targeting both MDM proteins simultaneously^21^. Consequently, the identification of molecules that target both the p53-MDM2 and p53-MDM4 pathways is imperative, notably in MDMX overexpressing cancer cells such as BC cells^22^,^23^.

Several studies have explored the developmental effects of the dual inhibitors of the p53-MDM2/X pathways, highlighting their prospects to confront cancer. However, the availability of such compounds is still limited and mostly focuses on *in vitro* and *in vivo* basic research, which requires further clinical evaluation^24^-^27^. In a previous study, we synthesized a cell-permeable dual-target MDM2/MDMX inhibitory protein, which contained the transactivator (TAT) peptide for transduction across membranes and the scaffold protein (thioredoxin A) displaying the MDM2/MDMX inhibitory peptide protein disulfide isomerase (pDI). This protein can bind to MDM2 and MDMX simultaneously and disrupt their interaction with p53. We further investigated the antitumor activity of this protein and demonstrated that it could reduce the viability of MCF-7 and ZR-75-30 BC cell lines and promote cell cycle arrest and apoptosis^28^. In addition, we validated the killing effect of MDM2/MDMX inhibitory protein on normal mammary epithelial cells in a dose-dependent manner. However, the function of the dual-target MDM2/MDMX inhibitory protein on DOX resistance of human BC has not yet been investigated. Based on the comprehensive role of p53 in drug resistance^10^,^11^, we investigated whether a dual-target MDM2/MDMX inhibitor could reverse DOX resistance in human breast cancer. We explored this hypothesis using two DOX-resistant BC cells with wild-type p53, and carried out functional studies using a nude mouse model and BC clinical specimens. We also investigated the possibility that the dual-target MDM2/MDMX inhibitory protein might reverse DOX resistance in human breast cancer through the activation of the transforming growth factor β-activated kinase 1 (TAK1)-binding protein 1 (TAB1) /TAK1/p38 mitogen-activated protein kinase (MAPK) signaling pathway.

## Materials and Methods

### Reagents

We synthesized the cell-permeable dual-target MDM2/MDMX inhibitory protein that could simultaneously disrupt the interactions of MDM2 and MDMX with p53. The process included construction of an expression vector, followed by gene expression, protein purification and protein refolding, as described in detail in previous studies. Afterwards, co-immunoprecipitation-western blot analysis showed the protein was able to be immunoprecipitated by anti-MDM2 and anti-MDMX antibodies, indicating that this protein is functional. Enzyme-linked immunosorbent assay (ELISA) proved that the recombinant dual-target MDM2/MDMX inhibitor strongly inhibited interaction of MDM2/MDMX with p53, which was in a dose-dependent manner^28^,^29^.

### Cell culture

The human breast adenocarcinoma cell line MCF-7 and MCF-7/DOX cells (DOX-resistant MCF-7 cells) were purchased from KeyGEN BioTECH (Nanjing, China). The human breast infiltrating duct carcinoma cell line ZR-75-30 was obtained from the Translational Medical Center of the Medical College of Xi'an Jiaotong University. ZR-75-30/DOX cells (DOX-resistant ZR-75-30 cells) were established from the corresponding sensitive cell line ZR-75-30 with a gradual increase of DOX (Topscience, Shanghai, China) concentration. The culture conditions were initially the same as those used for the ZR-75-30 cell line. Subsequently, DOX was added and the concentration was increased every two weeks with a medium exchange every two days. The DOX-resistant ZR-75-30 cell line was obtained following one year of culture. It is worth mentioning that both cell lines were wild-type p53.

All cell lines were cultured in RPMI-1640 medium (KeyGEN BioTECH) containing 10% fetal bovine serum (FBS, HyClone, Logan, UT, USA), 100 U/ml penicillin and 100 μg/ml streptomycin (Life Technologies, Grand Island, NY, USA) at 37ºC in a humidified atmosphere of 5% CO_2_. MCF‑7/ DOX and ZR-75-30/ DOX cells were cultured in media containing 1 μg/ml DOX to maintain the MDR phenotype, and prior to their use, the cells were maintained in drug-free media for at least two days.

### Western blot analysis

The cells were lyzed with ice-cold lysis buffer including protease and phosphatase inhibitors (Roche, Indianapolis, IN, USA). Following centrifugation at 4ºC for 20 min, the supernatants were collected and the protein concentration was determined using a bicinchoninic acid protein assay kit (Thermo Fisher Scientific, Waltham, MA, USA; 23227). Equivalent amounts of protein were fractionated by sodium dodecyl sulfate-polyacrylamide gels and transferred to a polyvinyl difluoride membrane (Millipore Corp., Boston, MA, USA). The membranes were blocked in 5% (w/v) non-fat milk at room temperature for 2 h, and incubated with primary antibodies at 4ºC overnight, followed by incubation with the corresponding HRP-conjugated secondary antibodies (Cell Signaling Technology, Boston, MA, USA; #7044; diluted at 1:5,000) at room temperature for 1 h. Finally, the blots were visualized with the enhanced chemiluminescence kit (Millipore Corp, Boston, MA) detection system according to the manufacturer's instructions. GAPDH (Proteintech, Wuhan, Hubei, China; HRP-60004, diluted at 1:5,000) was used as a loading control. The following primary antibodies were used: MDM2 (Abcam, Cambridge, UK; ab16895; 1:1,000), MDMX (Abcam; ab16058; 1:2,000); p53 (Wanleibio, Shenyang, Liaoning, China; WL02504; 1:500), P-gp (Abcam; ab129450; 1:2,000), MRP (Wanleibio; WL01027; 1:1,000), CyclinB1 (Wanleibio; WL01760; 1:1,000), cdc2 (Wanleibio; WL02373; 1:500), Bax (Wanleibio; WL03315; 1:1,000), Bcl-2 (Wanleibio; WL01556; 1:500), TAB1 (Abcam; ab76412; 1:2,000), TAK1 (Proteintech; 12330-2-AP, 1:2000), p38 MAPK (Proteintech; 14064-1-AP, 1:2,000).

### Sulforhodamine B (SRB) assay

The SRB assay was performed for the toxicity screening of cells using a range of widely used anticancer drugs. Following seeding of the cells at a density of 1×10^4^ cells/well in a 96-well plate and overnight incubation to allow adherence, the cells were treated with at least eight concentration gradients of DOX, cis-Dichlorodiamineplatinum (DDP), cyclophosphamide (CTX) and tamoxifen (TAM), respectively. The treatments were performed in triplicate and the maximum concentration used was 80 μg/ml. Dimethyl sulfoxide (DMSO; Sigma-Aldrich, St. Louis, MO, USA) was used as a vehicle control and a blank group containing blank phosphate buffer saline (PBS) was also used. Cell monolayers were fixed with 50% (w/v) trichloroacetic acid and stained with 0.4% (w/v in acetic acid) SRB (Sigma-Aldrich) solution for 30 min at room temperature. The stained cells were destained with 1% acetic acid to remove the excess dye. The protein-bound dye was dissolved in 10 mM Tris base solution for optical density (OD) determination at 540 nm using a microplate reader (Bio-Rad, Hercules, CA, USA). The rate of cell inhibition was calculated using the following formula: inhibition rate = [1 - (OD test/OD negative control)] × 100%. After plotting the dose-response curve, the half maximal inhibitory concentration (IC_50_) values of different drugs were calculated using SPSS software (Statistical Package for the Social Sciences, version 18.0, SPPS, Inc., Chicago, IL, USA) and the resistant fold (RF) value was determined by the following formula: IC_50_ (resistant cells)/IC_50_ (sensitive cells). Seeded cells were afterwards treated with different concentrations of MDM2/MDMX inhibitor (10, 20, 30, 40, 50, 60, 70 and 80 µg/ml) in a SRB assay in order to obtain the 10% inhibitory concentration (IC10) values as working concentrations. Moreover, the cells were exposed to various concentrations of DOX again after treatment with the MDM2/MDMX inhibitor to verify its reversal effect, and the reversal index (RI) value was determined by the following formula: IC_50_ (resistant cells before treatment with MDM2/MDMX inhibitor)/IC_50_ (resistant cells after treatment with MDM2/MDMX inhibitor).

### Plate clone formation assay

The cells were seeded in 6-well plates (3 × 10^2^ cells/well) and then treated with DOX and/or MDM2/MDMX inhibitor for 24 h. The cells were cultured in drug-free media for approximately 2 weeks until they were evaluated macroscopically and subsequently washed twice with PBS. The cells were then fixed with methanol and stained with 0.1% (w/v in PBS) crystal violet for 20 min. The colonies (more than 50 cells) were photographed and manually counted. The relative clone formation ability was calculated as follows: (mean experimental clone number/mean control clone number) ×100%.

### Flow cytometric analysis (FCM) to assess cell cycle progression and apoptosis

For cell cycle measurements, the cells were seeded in 6-well plates (3×10^5^ cells/well) that were separately treated with DOX and/or MDM2/MDMX inhibitor for 24 h. Adherent cells were collected, washed twice with cold PBS, and fixed with cold 70% ethanol at 4ºC overnight. Subsequently, the cells were stained with a solution that contained 25 μg/ml of propidium iodide with RNase A (50 μg/ml) for 30 min at 37ºC in the dark. The samples were analyzed with FCM (BD FACSCalibur; BD Biosciences, Franklin Lakes, NJ, USA). For measurements of cell apoptosis, the cells were double stained with Phycoerythrin (PE) Annexin V and 7-aminoactinomycin D (7-AAD) after harvesting and washing twice with PBS. Finally, stained cells were also detected by FCM according to the manufacturer's instructions (BD Biosciences; 559763).

### Rhodamine123 accumulation assay

The cells were seeded in 6-well plates (3×10^5^ cells/well), pre-treated with DOX and/or MDM2/MDMX inhibitor for 24 h and then exposed to 10 μM rhodamine123 (Sigma-Aldrich) for an additional 2 h in the dark. After harvesting and washing twice with PBS, the cells were detected with FCM with excitation and emission wavelengths of 485 and 535 nm, respectively and finally the cell-associated mean fluorescence intensity was determined^30^-^31^.

### Immunofluorescence staining

Following relevant treatment of the cells with the MDM2/MDMX inhibitor for 24 h, the cells were seeded in 24-well plates (5×10^4^ cells/well) with sterile glass coverslips and were subsequently fixed with 4% paraformaldehyde. Following blocking of the nonspecific binding sites with 5% (w/v) bovine serum albumin for 30 min, the slides were incubated with primary antibodies against P-gp (Abcam; 1:200) and MRP (Wanleibio; 1:200) at 4°C overnight. An additional reaction was carried out with Cy3-conjugated IgG (EK022, 1:200 dilution in PBS) at room temperature for 1 h. Finally, the cells were sealed with a fluorescence quenching sealing tablet containing 4', 6-diamidino-2-phenylindole (DAPI; Yeasen, Shanghai, China; 36308ES11). The images were captured with a fluorescence microscope (Nikon-Eclipse; Nikon, Tokyo, Japan; magnification: 200×).

### Xenograft model

Four-week-old female athymic BALB/c nude mice were purchased from the Shanghai Laboratory Animal Center of the Chinese Academy of Sciences and housed under specific-pathogen-free conditions in the Animal Center of the Medical College of Xi'an Jiaotong University. All animal experimental procedures were carried out according to the protocols approved by the Ethics Committee for Animal Experimentation of the Medical College of Xi'an Jiaotong University, in accordance with the National Institutes of Health Guide for the Care and Use of Laboratory Animals. MCF-7/DOX cells (5×10^6^ cells) were re-suspended in PBS, mixed with matrigel and were injected into the mammary fat pads of mice to establish mouse models of breast carcinoma *in situ*. When the average tumor size reached approximately 200 mm^3^, tumor-bearing mice were randomly divided into four groups (six in each group) and treated with the following regimens: (1) Normal saline (NS); (2) single treatment of DOX; (3) single treatment of MDM2/MDMX inhibitor; and (4) pre-treatment with MDM2/MDMX inhibitor followed by DOX. The epigenetic therapy used in the present study comprised i.v. administration of MDM2/MDMX inhibitor at 10 mg/kg, for the time period of d 1 to d 3. The DOX chemotherapy regimen was 2.5 mg/kg, iv, twice per week^32^. Tumor volume was estimated every other day using the formula: V (cm^3^) = a × b^2^/2 (where* a* was the largest diameter and *b* the smallest diameter). After several days, the mice were killed and tumor tissues were excised, weighed and fixed in 10% neutral buffered formalin for pathological analysis. The IRs were calculated according to the formula: inhibition rate (IR) = (1 ˗ mean tumor weight of the experimental group/mean tumor weight of the control group) ×100%.

### Immunohistochemistry (IHC)

The formalin-fixed, paraffin-embedded breast cancer tissues were sectioned (4 µm) and baked at 65ºC overnight. Following antigen retrieval in 10 mM of citrate buffer using a microwave, IHC staining was performed using a streptavidin-biotin peroxidase kit (Beijing Zhongshan Golden Bridge Biotechnology, China; SP-9001) according to the manufacturer's instructions. Subsequently, diaminobenzidine (Beijing Zhongshan Golden Bridge Biotechnology; ZLI-9018) was added to the sections, according to the manufacturer's instructions. Finally, the sections were counterstained with hematoxylin, dehydrated and mounted. Anti-P-gp polyclonal (Abcam; 1:200) or anti-MRP polyclonal antibodies (Wanleibio; 1:50) were applied and the slides were placed into a humid incubation chamber at 4ºC overnight. PBS was used as negative control instead of the primary antibody. Expression intensities were quantified as the sum of the integrated optical densities(IOD)/sum of the area of threshold pixels for all signals measured in each image using IPP 6.0 software (Media Cybernetics).

### SiRNA and transfection

The siRNA targeting TAB1 and the corresponding negative controls were designed and synthesized (GenePharma Co., Ltd., Shanghai, China). To knock down endogenous TAB1, the following target sequences were constructed in a siRNA vector: siRNA#1-TAB1-Homo-219: sense: 5′- GGAGUGAGAACAACUGCUUTT -3′, antisense: 5′- AAGCAGUUGUUCUCACUCCTT -3′; siRNA#2-TAB1-Homo-685: sense: 5′- GGAUGAGCUCUUCCGUCUUTT -3′, antisense: 5′- AAGACGGAAGAGCUCAUCCTT -3′; siRNA#3-TAB1-Homo-970: sense: 5′- GGAGAUUGCUGCGAUGAUUTT -3′, antisense: 5′- AAUCAUCGCAGCAAUCUCCTT -3′. A scrambled siRNA sequence was used as a negative control: 5′- UUCUCCGAACGUGUCACGUTT -3′, antisense: 5′- ACGUGACACGUUCGGAGAATT -3′. The cells were seeded in a 6-well plate (5 × 10^5^ cells/well) in medium without antibiotics. Following treatment for 24 h, the cells were transfected with double-stranded siRNA against TAB1 or nonspecific control siRNA using Lipofectamine 2000 (Thermo Fisher Scientific) according to the manufacturer's instructions. The efficiency of RNA interference was verified by western blot assays.

### Tissue microarrays (TMA)

A total of 3 TMA slides (Shanghai Outdo Biotech Co LTD, Shanghai, China), containing 70 breast cancer tissue samples (including 62 infiltrating ductal carcinoma and 8 intraductal carcinoma samples), were used to evaluate associations between MDM2/MDMX expression and TAB1 expression. The patients included 2 males and 68 females aged 30-89 years (mean age, 58.33 years). None of the patients had received anti-cancer treatment prior to tumor excision. All patients were classified according to the p-TNM staging system of the American Joint Committee on Cancer^33^ and the classification system of the World Health Organization^34^. The study was approved by the Medical Ethics Committee of Zhejiang Taizhou Hospital and the patients agreed to the use of their samples in scientific research.

We performed IHC staining of TMA slides with MDM2 (Abcam, ab16895; dilution, 1:100), MDMX (Abcam, ab154324; dilution, 1:100) and TAB1 (Abcam; ab76412; dilution, 1:200) antibodies by the procedure described in the IHC section. The Sinicrope scoring method^35^ was used to evaluate both the IHC staining intensity and the proportion of stained cells in each field. The intensity was classified as 0 for negative staining, 1 for weak staining, 2 for moderate staining, and 3 for strong staining. The scores were 0 for less than 5% stained cells, 1 for 6%-25% stained cells, 2 for 26%-50% stained cells, 3 for 51%-75% stained cells, and 4 for more than 75% stained cells. The proportion and staining intensities were multiplied to calculate an immuno-reactive score for each tumor specimen. For MDM2, MDMX and TAB1 expression, a final score of 0-2 indicated negative expression and of 3-12 positive expression.

### Statistical analysis

Statistical analysis was performed using the SPSS software. The data were expressed as mean ± SD. The differences between the two groups were analyzed using the student's t-test. The differences between three or more groups were analyzed using one-way ANOVA and the least-significant difference (LSD) test. The Chi-square test was used to analyze differences between clinicopathological variables. All statistical tests were two sided. A P value less than 0.05 (*P*<0.05) was considered to indicate a statistically significant difference.

## Results

### Multidrug resistance (MDR) characterization of MCF-7/DOX and ZR-75-30/DOX cells

**Figure 1:**
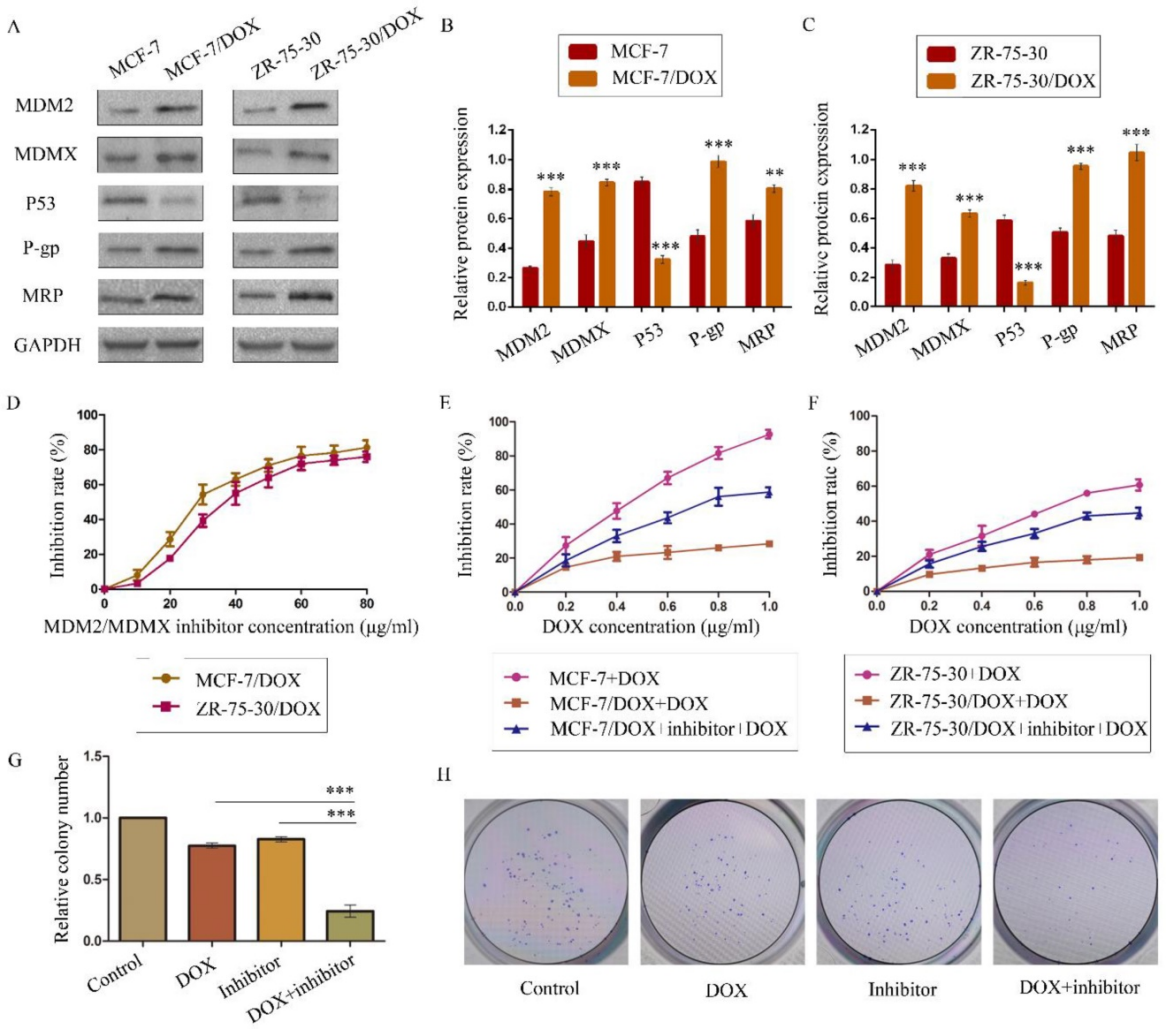
The expression levels of the p53 and MDR-related proteins in drug-resistant cells and the corresponding drug-sensitive cells were determined using western blot analysis. GAPDH was used as a loading control. The results are representative of three independent experiments (**A**) and quantified data are presented as the mean ± SD. *P<0.05, **P<0.01, ***P<0.001 vs. the corresponding drug-sensitive cells (**B, C**). The cells were treated with the indicated agents for 24 h, and cell survival was measured by an SRB assay in order to prove that MDM2/MDMX inhibitor enhanced DOX-induced cytotoxicity in DOX-resistant breast cells. The MDM2/MDMX inhibitor concentration-cell viability curve (**D**) was plotted to acquire the following working solution concentration. The growth curves of specific treatments are shown: MCF-7 with its homologous DOX-resistant MCF-7/DOX cells (**E**) and ZR-75-30 with its homologous DOX-resistant ZR-75-30/DOX cells (**F**) treated with DOX alone or in combination with the MDM2/MDMX inhibitor. The MDM2/MDMX inhibitor potentiated DOX-mediated inhibition of colony formation in MCF-7/DOX cells. The concentration of DOX in the colony formation assay was 0.08 µg/ml and that of the MDM2/MDMX inhibitor was 9.58 µg/ml. The quantified data (**G**) were presented as the mean ± SD and representative charts (**H**) are shown. *P<0.05, **P<0.01, ***P<0.001 (n = 3) vs. the DOX only group or the MDM2/MDMX inhibitor group.

The DOX resistance and the MDR status of MCF-7/DOX and ZR-75-30/DOX cells were determined using concentration-cell viability curves plotted through a SRB assay. The RFs for DOX were higher than 1 (> 1) for both cell types, confirming their DOX resistance. MDR has been previously reported as the underlying reason for drug resistance^9^,^30^. Thus, we determined the IC_50_ values of the widely used anticancer drugs DDP, CTX and TAM in order to explore the MDR phenotype of the two drug-resistant cells. The IC_50_ values for these drugs in drug-resistant cells increased dramatically compared with homologous drug-sensitive cells (Table 1). All the RFs were higher than 1 (> 1), demonstrating that both drug-resistant cells exhibited MDR phenotypes and were suitable cell lines that could be used to evaluate the effects of the MDM2/MDMX inhibitor on DOX resistance in BC. In addition, overexpression of MDM2, MDMX, P-gp and MRP and low expression of p53 were detected in the resistant cell lines compared with the parental cells (Figure 1A-1C). These results further provided evidence that DOX resistance was related to the expression of MDR-related proteins.

### The MDM2/MDMX inhibitor could reverse the DOX resistance of drug-resistant BC cells

We confirmed the antitumor activity of the MDM2/MDMX inhibitor in a previous study^28^. In the present study, our objective was to investigate the capacity of this protein to reverse DOX resistance, and not its antitumor activity. SRB assay data indicated that this recombinant protein and DOX both inhibited the viability of BC cells in a dose-dependent manner. We plotted protein concentration-cell viability curves (Figure 1D) and acquired the IC10 values of the protein as the working concentrations in the following experiments in order to avoid tumor cell death due to the protein's toxicity^30^,^36^-^37^. The IC10 values of the MDM2/MDMX inhibitor in MCF-7/DOX and ZR-75-30/DOX cells were 9.58 ± 1.35 µg/ml and 13.70 ± 1.12 µg/ml, respectively.

To evaluate the reversal effect of MDM2/MDMX inhibitor on the DOX resistance of MCF-7/DOX and ZR-75-30/DOX cells, the DOX concentration-cell viability curves were plotted. MCF-7/DOX and ZR-75-30/DOX cells exhibited apparent resistance to DOX compared to MCF-7 and ZR-75-30 cells (Figure 1E and 1F). However, treatment by the MDM2/MDMX inhibitor decreased the IC_50_ values of DOX from 6.58 ± 1.50 µg/ml and 16.88 ± 5.64 µg/ml to 0.72 ± 0.08 µg/ml and 1.21 ± 0.17 µg/ml in MCF-7/DOX and ZR-75-30/DOX cells, respectively. The RIs of MCF-7/DOX and ZR-75-30/DOX cells were 9.15 and 13.92, respectively. This indicated that the MDM2/MDMX inhibitor significantly reversed the resistance of the cells to DOX. In addition, we used the IC10 values of DOX as the working concentrations in the following experiments. This corresponded to 0.08 ± 0.01 µg/ml in MCF-7/DOX cells and 0.20 ± 0.07 µg/ml in ZR-75-30/DOX cells.

### The MDM2/MDMX inhibitor in combination with DOX could inhibit the proliferation of drug-resistant BC cells

A colony formation assay was performed to study the anti-proliferative effects of the combination of the MDM2/MDMX inhibitor and DOX in MCF-7/DOX cells. The combination of the MDM2/MDMX inhibitor and DOX inhibited the proliferation of MCF-7/DOX cells significantly, with a colony formation rate of 24.33 ± 5.03% detected for this group, which was significantly lower than the DOX group (Figure 1G and 1H, 77.33 ± 2.08%; *P*<0.001) and the MDM2/MDMX inhibitor group (82.67 ± 2.08%; *P*<0.001). Furthermore, single treatments of DOX (*P*<0.001) and/or the MDM2/MDMX inhibitor (*P*<0.001) could also inhibit the growth of MCF-7/DOX cells significantly, whereas no significant difference was noted between them (*P*=0.055).

### The MDM2/MDMX inhibitor in combination with DOX induces cell cycle arrest in the drug-resistant BC cells

**Figure 2:**
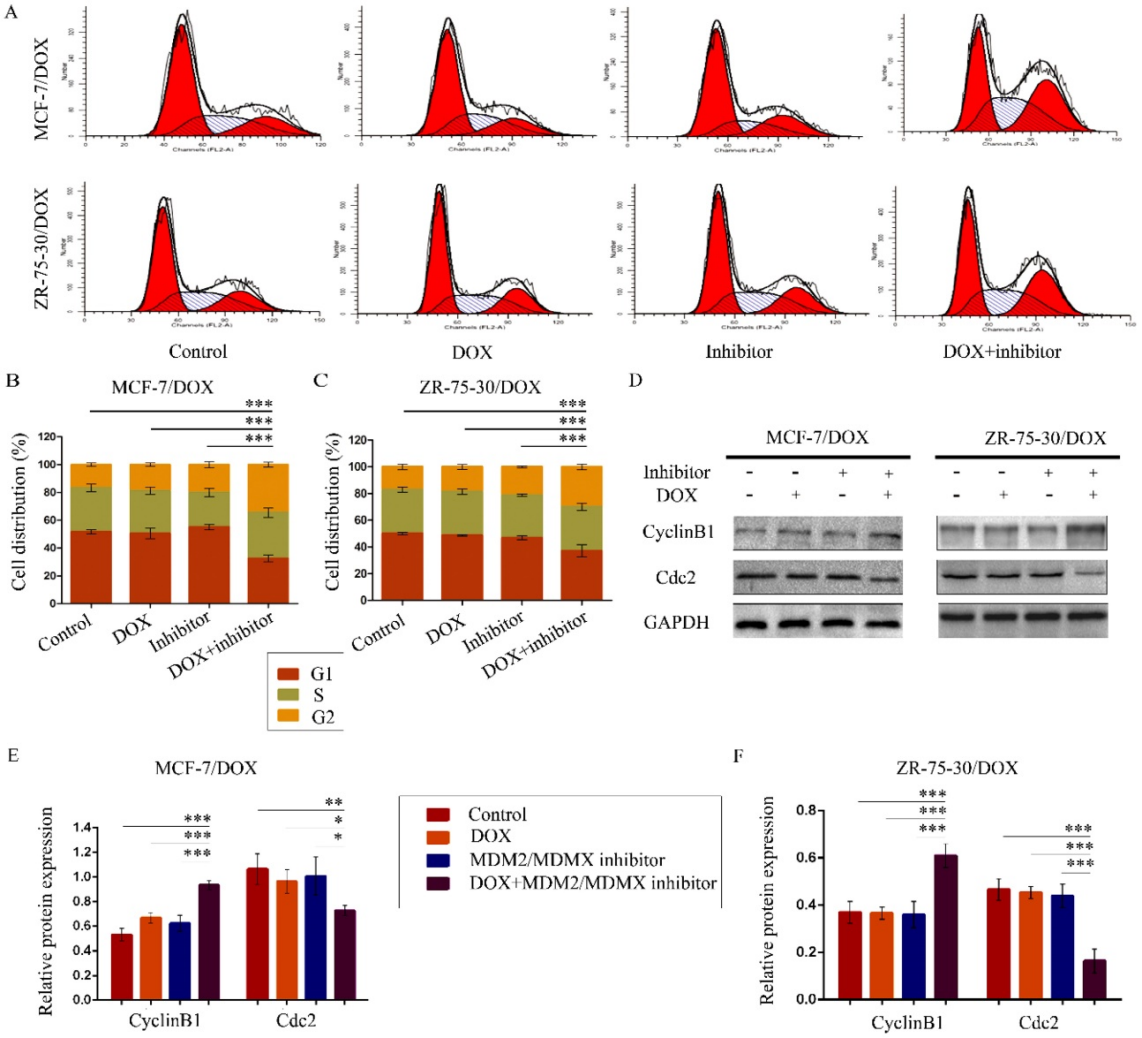
The MDM2/MDMX inhibitor in combination with DOX induces cell cycle arrest in the drug-resistant breast cancer cells. The cells were treated with the indicated agents for 24 h, and the cell cycle distribution was detected using FCM with PI staining. The concentration levels of each agent were used as follows: DOX 0.08 µg/ml in MCF-7/DOX and 0.20 µg/ml in ZR-75-30/DOX, MDM2/MDMX inhibitor 9.58 µg/ml in MCF-7/DOX and 13.70 µg/ml in ZR-75-30/DOX. The representative charts (**A**) and quantified data (**B, C**) of the cell cycle are shown. Protein expression was examined by western blot analysis after lysing the cells, and GAPDH was used as a loading control. The representative charts (**D**) and quantified data (**E, F**) and of western blot analysis are also shown. The values presented are indicative of the mean ± SD for each group. *P<0.05, **P<0.01, ***P<0.001 (n = 3) vs. the control group, the DOX only group or the MDM2/MDMX inhibitor group.

To evaluate the effects of the MDM2/MDMX inhibitor in combination with DOX on the cell cycle of drug-resistant BC cells, the cell cycle distribution and the related proteins were detected by FCM and western blot analysis, respectively. The representative cell cycle results were shown in Figure 2A. Co-treatment with the MDM2/MDMX inhibitor and DOX significantly increased the cell population at the G2/M phase in MCF-7/DOX (34.5 ± 1.8%) and ZR-75-30/DOX (29.95 ± 2.00%) cell lines, which were significantly higher than the control (*P*<0.001), the DOX (*P*<0.001) and the MDM2/MDMX inhibitor groups (*P*<0.001) (Figure 2B and 2C). It is important to note that the single treatment of the cells with the MDM2/MDMX inhibitor could significantly block the cell cycle progression of MCF-7/DOX (*P*=0.039) and ZR-75-30/DOX (*P*=0.017) cells to the G2/M phase, although single treatment with DOX did not exhibit this effect. Co-treatment with the MDM2/MDMX inhibitor and DOX increased the levels of the G2/M-phase dominant protein cyclin B1 and decreased the levels of cdc2, which provided further evidence that the MDM2/MDMX inhibitor in combination with DOX could induce G2/M arrest of MCF-7/DOX and ZR-75-30/DOX cells (Figure 2D-2F).

### The MDM2/MDMX inhibitor in combination with DOX promotes apoptosis in the drug-resistant BC cells

**Figure 3:**
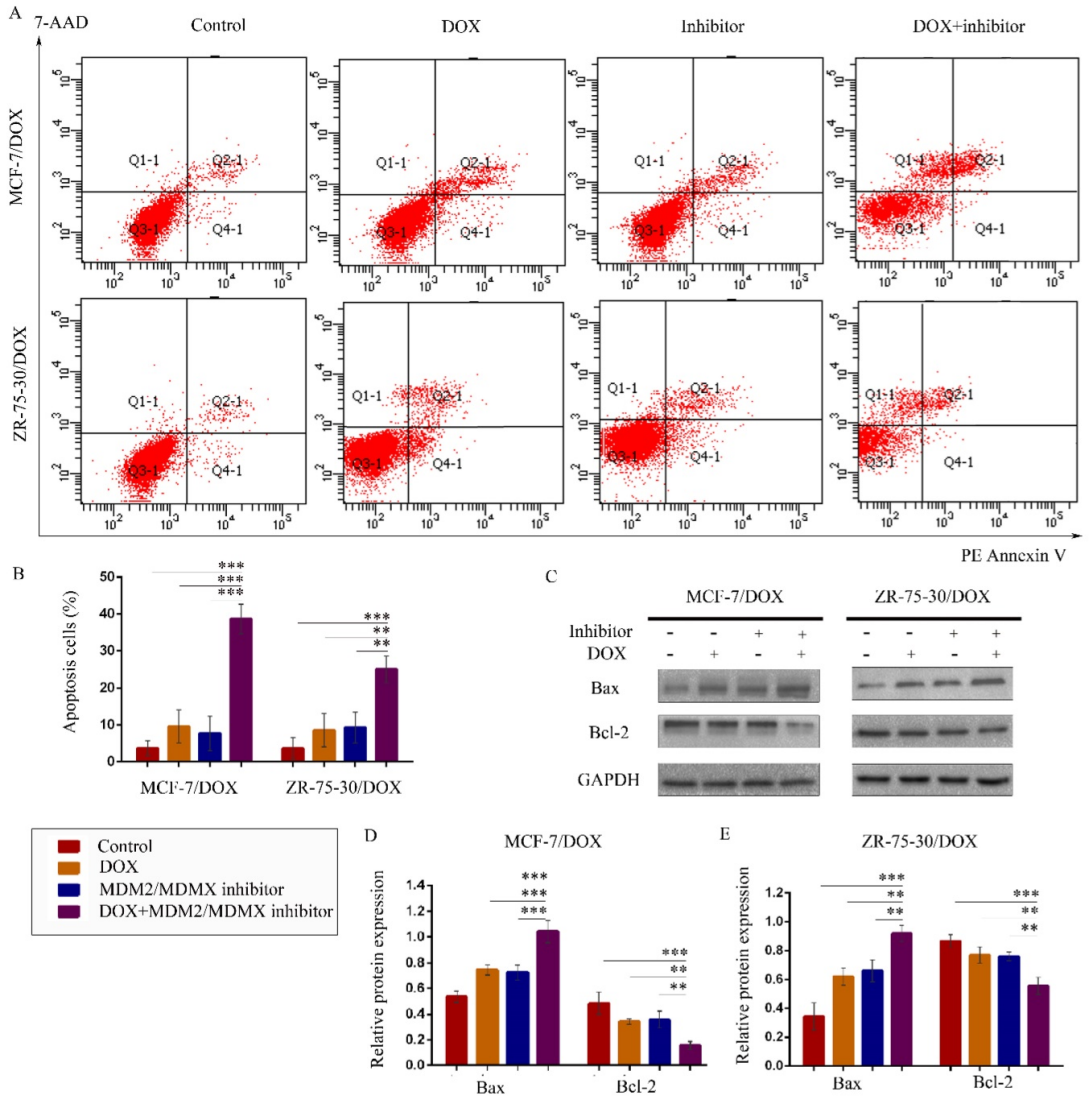
The MDM2/MDMX inhibitor in combination with DOX induces apoptosis in the drug-resistant breast cancer cells. The cells were treated with the indicated agents for 24 h, and apoptosis was detected by FCM with PE Annexin V /7-AAD staining. The proportions of PE Annexin V+/7-AAD˗ and PE Annexin V+/7-AAD+ cells indicated the early and late stages of apoptosis. The concentration levels of each agent were used as follows: DOX 0.08 µg/ml in MCF-7/DOX and 0.20 µg/ml in ZR-75-30/DOX, MDM2/MDMX inhibitor 9.58 µg/ml in MCF-7/DOX and 13.70 µg/ml in ZR-75-30/DOX. The representative charts (**A**) and quantified data (**B**) of apoptosis are shown. Protein expression was examined by western blot analysis following cell lysis, and GAPDH was used as a loading control. The representative charts (**C**) and quantified data (**D, E**) of the western blot analysis are also shown. The values presented are representative of the mean ± SD for each group. *P<0.05, **P<0.01, ***P<0.001 (n = 3) vs. the control group, the DOX and/or the MDM2/MDMX inhibitor groups.

To explore whether treatment with the MDM2/MDMX inhibitor in combination with DOX promotes apoptosis, drug-resistant cells were stained with PE Annexin V and 7-AAD, followed by FCM analysis. The representative evaluation of apoptosis is shown in Figure 3A. Co-treatment with the MDM2/MDMX inhibitor and DOX resulted in an elevated number of apoptotic cells in both resistant cell lines (38.67 ± 4.04% and 25.00 ± 3.61%, respectively) compared with the control group (*P*<0.001), the DOX group (*P*<0.01) and the MDM2/MDMX inhibitor group (*P*<0.01) (Figure 3B). Further analysis using western blotting revealed that co-treatment with the MDM2/MDMX inhibitor and DOX decreased Bcl-2 protein levels and increased Bax protein levels (Figure 3C-3E). Taken collectively, the data indicated that the MDM2/MDMX inhibitor in combination with DOX could promote apoptosis in MCF-7/DOX and ZR-75-30/DOX cells.

### The MDM2/MDMX inhibitor in combination with DOX reduces rhodamine123 efflux in drug-resistant BC cells

**Figure 4:**
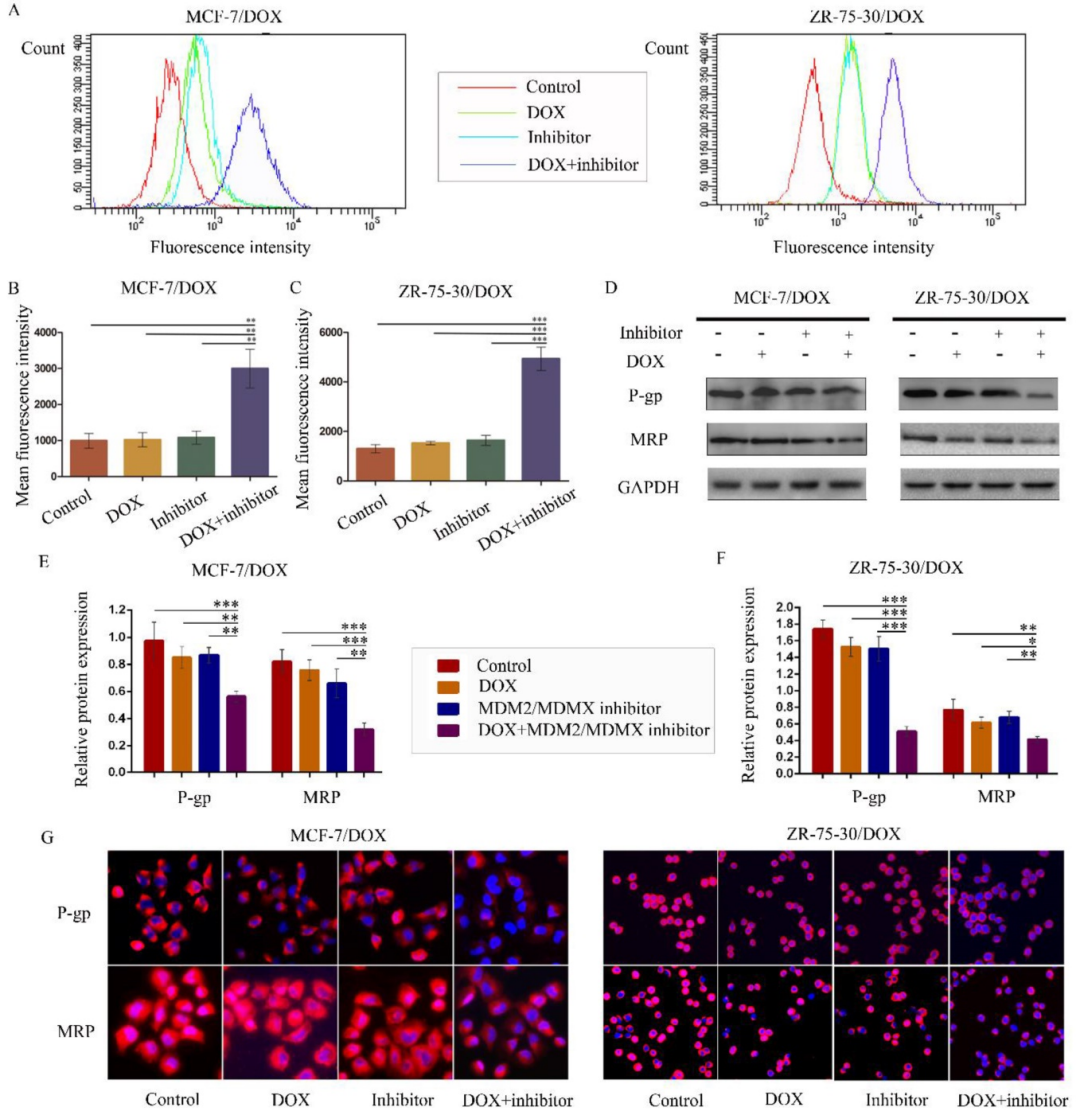
The MDM2/MDMX inhibitor in combination with DOX reduces rhodamine 123 efflux in drug-resistant breast cancer cells. The cells were treated with the indicated agents for 24 h, and intracellular rhodamine 123 levels were measured to explore the transporter activity of the membrane pump protein P-gp. The concentration levels of each agent were used as follows: DOX 0.08 µg/ml in MCF-7/DOX and 0.20 µg/ml in ZR-75-30/DOX, MDM2/MDMX inhibitor 9.58 µg/ml in MCF-7/DOX and 13.70 µg/ml in ZR-75-30/DOX. The representative charts (**A**) and quantified data (**B, C**) of the rhodamine 123 assay are shown. The expression levels of the proteins investigated were examined by western blot analysis, and GAPDH was used as a loading control. The representative charts (**D**) and quantified data (**E, F**) of the western blot analysis are also shown. The values presented are indicative of the mean ± SD for each group. *P<0.05, **P<0.01, ***P<0.001 (n = 3) vs. the control group, the DOX group and/or the MDM2/MDMX inhibitor group. Immunofluorescence staining was also used in drug-resistant breast cancer cells to confirm the expression of the proteins investigated. The representative charts (**G**) of three independent experiments are shown.

To examine whether the antagonism of DOX resistance by the MDM2/MDMX inhibitor was attributed to the inhibition of the transporter activity of the membrane pump protein P-gp, we measured the intracellular levels of the P-gp substrate rhodamine 123. Representative rhodamine 123 results were shown in Figure 4A. The mean fluorescence intensity (MFI) represented the intracellular levels of rhodamine 123. Treatment with the MDM2/MDMX inhibitor in combination with DOX significantly increased MFIs in both MCF-7/DOX and ZR-75-30/DOX cells compared with the control groups (*P*<0.01), the DOX groups (*P*<0.01) and the MDM2/MDMX inhibitor groups (*P*<0.01), suggesting that the MDM2/MDMX inhibitor was able to reduce the drug efflux function of P-gp with the combined action of DOX (Figure 4B and 4C). By western blot analysis, we also demonstrated that co-treatment with the MDM2/MDMX inhibitor and DOX decreased P-gp and MRP protein levels (Figure 4D-F). Moreover, this treatment resulted in attenuated P-gp and MRP expression levels that were detected in the cell membrane by immunofluorescence staining. These effects were observed in the co-treatment groups in both drug-resistant BC cells (Figure 4G).

### The MDM2/MDMX inhibitor reverses the DOX resistance of BC in vivo

**Figure 5:**
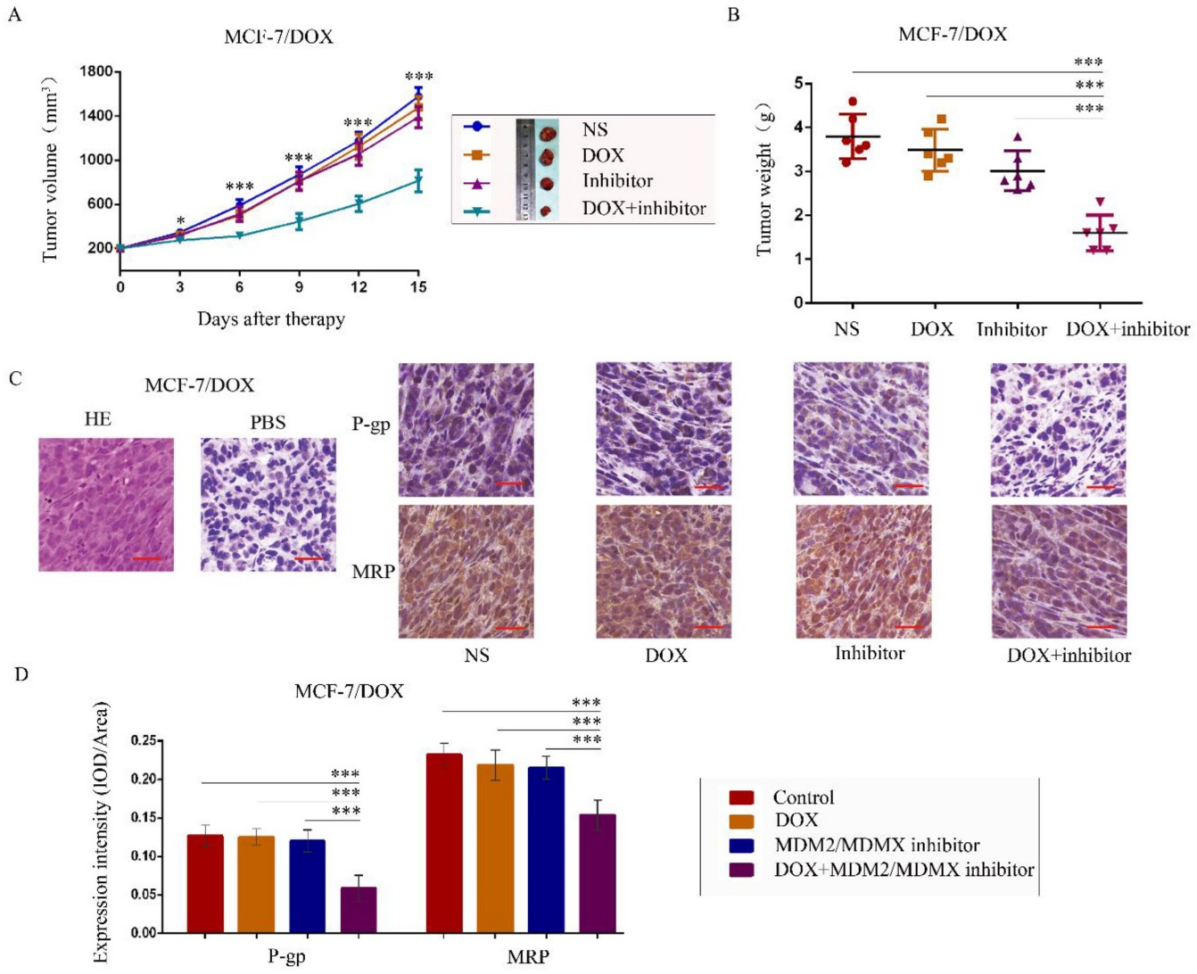
The MDM2/MDMX inhibitor can reverse DOX resistance of breast cancer in nude mice. Each mouse group was injected into the mammary fat pad with MCF-7/DOX cells (5×10^6^ cells). The cells were re-suspended in PBS and mixed with matrigel. When the tumors reached the approximate size of 200 mm^3^, tumor-bearing mice were randomly divided into four groups (six in each group) and treated with the following regimens: Normal saline; DOX (2.5 mg/kg, iv, twice per week); MDM2/MDMX inhibitor (10 mg/kg, iv, d1-3); and the combination of MDM2/MDMX inhibitor and DOX. The body weights of mice and the tumor volumes were recorded. The tumor volume and tumor weight quantified data (**A, B**) is shown. Representative hematoxylin-eosin staining (H&E) and immunohistochemical P-gp and MRP staining of breast cancer xenografts (**C**), and expression intensity calculated as IOD/Area (**D**) are also presented. Bars: 50 μm. The values presented are indicative of the mean ± SD for each group. *P<0.05, **P<0.01, ***P<0.001 (n = 6) vs. the control group, the DOX group or the MDM2/MDMX inhibitor group.

To further explore the antitumor effects of the co-treatment with the MDM2/MDMX inhibitor and DOX *in vivo*, MCF-7/DOX *in situ* xenograft models were generated in nude mice. When the subcutaneous tumor volume reached approximately 200 mm^3^, the mice were assigned to different groups according to different treatments. The transplanted tumor volumes in the co-treatment group of MCF-7/DOX models were significantly lower than those in the NS (*P*<0.001), DOX (*P*=0.004) and MDM2/MDMX inhibitor groups (*P*=0.011) since day 3 until mice executed (Figure 5A). On the last day of observation (day 15), the IRs of the DOX alone group, MDM2/MDMX inhibitor alone group and co-treatment group were 8.33%, 20.61%, and 57.89%, respectively compared to the NS groups (Figure 5B). No loss of body weight was detected among mice in the combination group, suggesting that the regimen of the combined treatment at the indicated dose did not cause toxicity *in vivo*. In addition, hematoxylin-eosin and immunohistochemical (IHC) staining of tumor tissue sections from the MCF-7/DOX model showed decreased P-gp and MRP levels in the tumor cell membrane in co-treatment group tumor tissues (Figure 5C and 5D).

### The MDM2/MDMX inhibitor reversed the DOX resistance in BC cells by activating the TAB1/TAK1/p38 MAPK signaling pathway

Having shown that the MDM2/MDMX inhibitor could reverse DOX resistance of BC *in vitro* and *in vivo*, the next step was to explore its associated mechanism of action. Mitogen-activated protein kinases (MAPKs) mediate a wide variety of cellular behaviors in response to extracellular stimuli^38^. One of the main subgroups of this class of enzymes, the p38 MAPK, has been implicated in a wide range of complex biological processes, such as cell proliferation, cell differentiation, cell death, cell migration and invasion^39^. TAB1, an activator of TAK1 and of p38α, was recently reported to associate with the E3 ligase and inhibit the activity of MDM2 against p53 and MDMX. It was previously established that TAB1 interacted with MDM2 and that TAB1 ablation attenuated p53 activation that resulted from knockdown of MDM2^40^.

**Figure 6:**
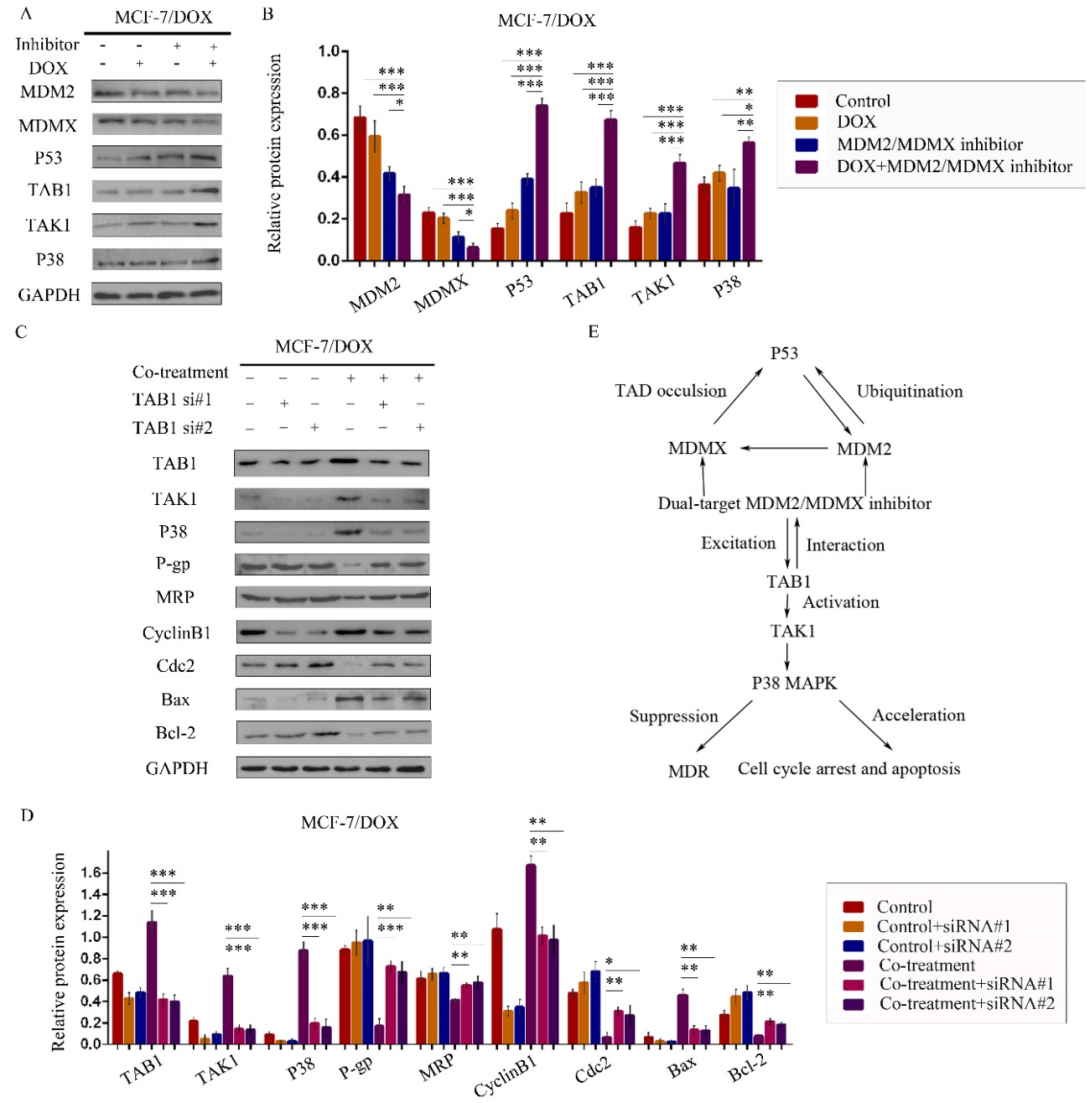
The MDM2/MDMX inhibitor reverses DOX resistance of breast cancer cells via the activation of the TAB1/TAK1/p38 MAPK pathway. The cells were treated with the indicated agents for 24 h. The concentration levels of each agent were used as follows: DOX 0.08 µg/ml and MDM2/MDMX inhibitor 9.58 µg/ml in MCF-7/DOX. Western blot analysis confirmed that co-treatment with the MDM2/MDMX inhibitor and DOX could lead to TAB1/TAK1/p38 MAPK overexpression in the drug-resistant breast cancer cells. The representative charts (**A**) and quantified data (**B**) are shown. *P<0.05, **P<0.01, ***P<0.001 (n = 3) vs. the control group, the DOX group or the MDM2/MDMX inhibitor group. Subsequently, cells were transfected with siRNA#1 and siRNA#2 with appropriate agent dosages at 1:2 (siRNA: Lipofectamine 2000; μg:μg) following co-treatment with MDM2/MDMX inhibitor and DOX for 24 h. Western blot analysis including the representative charts (**C**) and quantified data (**D**) of TAB1, TAK1, p38 MAPK, P-gp, MRP, CyclinB1, cdc2, Bax and Bcl-2 are shown. GAPDH was used as a loading control. *P<0.05, **P<0.01, ***P<0.001 (n = 3) vs. co-treatment group transfected with siRNA#1 or siRNA#2. The values presented are the mean ± SD for each group. Mutual regulations between the molecules are shown (**E**).

We therefore speculated that the MDM2/MDMX inhibitor could act through the TAB1/TAK1/p38 MAPK signaling pathway. Subsequently, western blot analysis was applied to confirm that co-treatment with the MDM2/MDMX inhibitor and DOX could lead to TAB1, TAK1 and p38 MAPK overexpression in the MCF-7/DOX cells (Figure 6A and 6B), demonstrating that the reversal of DOX resistance by the MDM2/MDMX inhibitor was actually related to the TAB1/TAK1/p38 MAPK pathway.

To investigate the interaction between the MDM2/MDMX inhibitor and the TAB1/TAK1/p38 MAPK pathway, we used small interfering RNA (siRNA) to knockdown TAB1 in both DOX-resistant cells. Subsequent western blot analysis revealed that siRNA-mediated TAB1 knockdown attenuated the effect of the MDM2/MDMX inhibitor on the expression of certain key proteins (Figure 6C and 6D). These results demonstrated that the MDM2/MDMX inhibitor reversed the DOX resistance of BC cells by activating the TAB1/TAK1/p38 MAPK pathway. Mutual regulations between the molecules are shown in Figure 6E.

**Figure 7:**
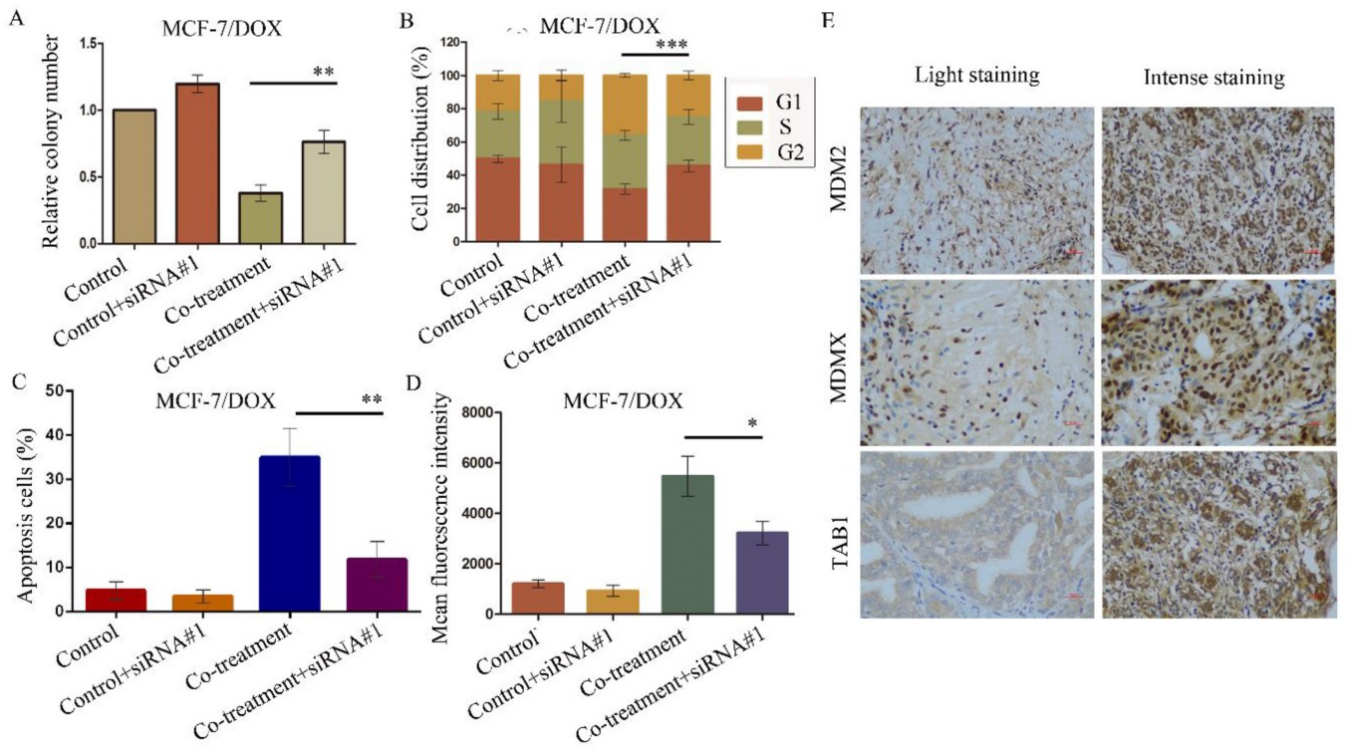
SiRNA#1-mediated TAB1 knockdown attenuates cellular function changes caused by co-treatment with MDM2/MDMX inhibitor and DOX in MCF-7/DOX cells. MCF-7/DOX cells were transfected with siRNA#1 following co-treatment with MDM2/MDMX inhibitor and DOX with appropriate agent dosages at 1:2 (siRNA: Lipofectamine 2000; μg:μg). The concentration levels of the agents that were used were 0.08 µg/ml for DOX and 9.58 µg/ml for the MDM2/MDMX inhibitor. Quantified data (**A-D**) of plate clone formation assay, cell cycle analysis, apoptotic assays and rhodamine123 efflux analysis are shown. The values presented are the mean ± SD for each group. *P<0.05, **P<0.01, ***P<0.001 vs. co-treatment with MDM2/MDMX inhibitor and DOX groups (n=3). MDM2, MDMX and TAB1 expression in 70 human breast cancer tissues were detected by IHC staining. Representative images for MDM2, MDMX and TAB1 are shown in (**E**). Magnification: 400×.

The aforementioned data were verified by the evaluation of the cellular function caused by the siRNA-mediated TAB1 knockdown in MCF-7/DOX cells. The knockdown of TAB1 in the cells of the co-treatment group displayed increased clone formation (*P*=0.003) (Figure 7A), reduced G2/M arrest (*P*<0.001) (Figure 7B), reduced cell apoptosis (*P*<0.001) (Figure 7C) and enhanced rhodamine123 efflux (*P*=0.014) (Figure 7D), which further demonstrated the role of the TAB1/TAK1/p38 MAPK pathway in reversing DOX resistance.

### MDM2, MDMX and TAB1 expression in BC tissues was associated with patient clinicopathological characteristics

We detected MDM2, MDMX and TAB1 expression in 70 breast cancer tissues with IHC staining. MDM2 was expressed in both the cytoplasmic and nuclear regions, whereas MDMX was expressed in the nuclei and TAB1 was expressed in the cytoplasm (Figure 7E). The associations between the expression levels of these proteins and various patient clinicopathological characteristics are shown in Table 2. MDM2 and TAB1 expression levels were associated with histological grading, whereas MDM2 and MDMX expression levels were associated with tumor size.

### MDM2/MDMX expression was positively correlated with TAB1 expression in BC tissues

Finally, the correlations between MDM2/MDMX and TAB1 expression levels were explored. The results indicated that high MDM2 expression was positively associated with weak TAB1 expression in the cytoplasm (*P*=0.001) and consistently, MDMX over-expression was also related to low expression of TAB1 in BC patients (*P*<0.001), which confirmed that the MDM2/MDMX inhibitor acted via the TAB1/TAK1/p38 MAPK pathway in the patient specimens examined.

## Discussion

The incidence rate of BC remains the highest among women with malignant tumors. Chemotherapy plays a crucial role in the systemic treatment of this disease, but more than 90% of cancer patients do not fully respond to chemotherapy agents^41^. Dual-target MDM2/MDMX inhibitor is a promising anti-cancer strategy and has been studied in a variety of tumors^24^-^27^. However, most of the previous studies focused on small molecule inhibitor, which was characterized by easy hydrolysis, poor stability, short-term half-life and poor permeability. Therefore, their studies were limited to preliminary cell function verification and difficult to carry out subsequent animal and clinical exploration. We optimized their defects and synthesized cell-permeable dual-target MDM2/MDMX inhibitory protein, which contained the TAT peptide for transduction across cell membrane and thioredoxin A for displaying the MDM2/MDMX inhibitory pDI. Our present study firstly confirmed the effect of the MDM2/MDMX inhibitory protein on reversing DOX resistance of BC cells with several novelty cell function experiments, such as SRB colorimetric assay and rhodamine 123 efflux assay. Also, we verified the *in vivo* anti-tumor effect of the protein with a human breast cancer orthotopic transplantation tumor model established in nude mice. Finally, we firstly displayed MDM2, MDMX and TAB1 expression in the BC tissue sample chips containing 70 cases and explored the relationship between their expression and patient clinicopathological characteristics. Specifically, the selection of the cell lines used in the present study was well justified. MCF-7 and ZR-75-30 cells both contain a wild-type p53 status, which mainly determines the response of the cells to genotoxic modalities.

The MDM2/MDMX inhibitor and DOX may block the cell cycle of drug-resistant breast cancer cells at the G2/M arrest via the induction of p21^42^,^43^. It is interesting to note that the single treatment of the MDM2/MDMX inhibitor could also significantly block the cell cycle progression of the two drug-resistant BC cells to the G2/M phase, although DOX could not, which suggested that the effects on the cell cycle were attributed to the action of the MDM2/MDMX inhibitor. P38α was activated by TAB1 through direct binding and was able to phosphorylate p53 N-terminal sites, leading to selective induction of p53 targets. It could be assumed that activated TAB1 caused by co-treatment with MDM2/MDMX inhibitor and DOX, altered the p38α status of the cells, which in turn phosphorylated p53 to mediate an apoptotic response. Concomitantly, TAB1 modulated the cellular levels of MDMX and facilitated MDMX mitochondrial localization, which contributed to the p53-mediated intrinsic apoptotic response.

A previous study has highlighted that TAB1 levels exert little or no effect on cell cycle arrest or cell death caused by various genotoxic assaults, with the exception of cisplatin^40^. However, the present results indicated that siRNA-mediated TAB1 knockdown in MCF-7/DOX cells attenuated G2/M arrest and apoptosis caused by co-treatment with the MDM2/MDMX inhibitor and DOX. These findings implied that the MDM2/MDMX inhibitor increased the potency of DOX via the TAB1/TAK1/p38 MAPK pathway. Taken collectively with previous reports, the data suggest a positive feedback loop involving TAB1, MDM2, MDMX and p53. The current results propose a new role for the MDM2/MDMX inhibitor as a potential strategy to treat cancer. However, an understanding of the molecular mechanism that mediates the MDM2/MDMX-TAB1 interaction is required and will be the subject of further studies.

We used siRNA to knockdown TAB1 in order to explore whether TAB1 knockdown could attenuate the effect of MDM2/MDMX inhibitor. However, it is inadequate to prove the mechanism of TAB1/TAK1/p38 MAPK pathway. Therefore, we plan to purchase the TAB1 overexpressed lentiviral from GeneChem Co., Ltd. (Shanghai, China) which is available and continued to explore whether TAB1 overexpression could enhance the effect of MDM2/MDMX inhibitor. In addition, we haven't realized how MDM2/MDMX inhibitor act on TAB1 in detail. Is it direct action or indirect effect? We need to carry out a co-immunoprecipitation-western blot analysis using a co-immunoprecipitation kit (Genmed, Shanghai, China) to explore the inter-reaction of MDM2/MDMX and TAB1. Recent studies have shown that miR-134 affects the sensitivity of breast cancer cells to DOX by down-regulating the expression level of MRP protein^44^. We intend to observe the regulation of miR-134 on TAB1 pathway in the future, and explored whether the effect of MDM2/MDMX inhibitor on reversing drug resistance was related to miR-134 regulation.

A clinical experiment was performed to investigate the aforementioned hypothesis. We demonstrated that MDM2 and TAB1 expression was associated with histological grading, and that MDM2/MDMX expression was associated with tumor size. Histological grading and tumor size are two variables that are closely related to prognosis. Thus we speculated that MDM2/MDMX/TAB1 expression might also correlate with patient outcomes. However, the lack of follow-up data was one of the limitations to our study. We only evaluated MDM2/MDMX and TAB1 expression by IHC and collected the clinicopathological characteristics of 70 breast cancer patients in order to explore the correlations between MDM2/MDMX and TAB1 expression levels. The follow-up studies of breast cancer patients that could be used to monitor their survival would help to elucidate the mechanisms and patient outcomes related to MDM2/MDMX inhibition. We searched The Cancer Genome Atlas (TCGA) but found no differences between MDM2/MDMX/TAB1 expression and breast cancer prognosis in their data, which may need to be tested in a larger sample.

Immune checkpoint inhibitors have demonstrated desirable anticancer effects, and the response rates for single agent immune checkpoint inhibitors in solid malignancies range from 20% to 40%^45^-^47^. However, a recent study reported on patients with MDM2/MDMX amplification, who exhibited poor clinical outcomes and significantly increased rates of tumor growth following treatment with single-agent checkpoint (PD-1/PD-L1) inhibitors^48^. This suggested that the targeting of both MDM2 and MDMX could be a promising therapeutic strategy to ensure that BC patients benefit from PD-1/PD-L1 inhibitors. Breast cancer patients could therefore be treated with both MDM2/MDMX inhibitors and PD-1/PD-L1 inhibitors to achieve an optimal therapeutic effect. Further investigations on this topic are urgently needed.

In conclusion, our results indicated that the MDM2/MDMX inhibitor reversed DOX resistance of human BC by activating the TAB1/TAK1/p38 MAPK pathway, suggesting that the MDM2/MDMX inhibitor in combination with chemotherapy may provide a novel therapeutic strategy for cancer treatment. However, a prospective randomized study of MDM2/MDMX inhibitor versus standard therapy would be required in order to lead to an unbiased conclusion of comparative progression rates between DOX resistance and MDM2/MDMX inhibition.

## Figures and Tables

**Table 1:** Determination of the IC_50_ values of different anticancer drugs

Drugs	IC_50_ (µg/ml)		RF	IC_50_ (µg/ml)		RF
	MCF-7	MCF-7/DOX		ZR-75-30	ZR-75-30/DOX	
DOX	0.37±0.03	6.58±1.50	17.59	0.70±0.03	16.88±5.64	24.11
DDP	3.01±0.59	10.77±1.91	3.59	4.78±1.77	9.28±1.15	1.94
CTX	5.59±1.28	37.89±3.94	6.79	2.26±0.83	15.26±1.56	6.74
TAM	4.25±0.29	12.19±2.64	2.87	3.45±0.23	17.65±2.36	5.12

**Notes:** IC_50_ values of anticancer drugs were determined in MCF-7 and ZR-75-30 cells and the corresponding drug-resistant MCF-7/DOX and ZR-75-30/DOX cells. The cells were treated with various concentrations of DOX, DDP, CTX and TAM for 24 h. The IC_50_ values and the RFs were evaluated. The data are expressed as the mean ± SD of three independent experiments. **Abbreviations:** IC_50_, half maximal inhibitory concentration; DOX, doxorubicin; DDP, cis-Dichlorodiamineplatinum; CTX, cyclophosphamide; TAM, tamoxifen; RF, resistant fold.

**Table 2:** Associations between MDM2/MDMX/TAB1 expression levels and the clinicopathological characteristics of breast cancer patients

Variables	No.	MDM2				MDMX				TAB1		
		Weak	High	*P*-value		Weak	High	*P*-value		Weak	High	*P*-value
**Age, years**												
<60	40	12	28	0.153		18	22	0.800		21	19	0.585
≥60	27	4	23			13	14			16	11	
**Grade**												
I/II	28	11	17	0.012^a^		14	14	0.644		11	17	0.013^a^
III	34	4	30			15	19			24	10	
**Stage**												
I/II	49	12	37	0.761		23	26	0.753		25	24	0.227
III/IV	21	4	17			9	12			14	7	
**LM**												
Negative Positive	33 34	11 5	22 29	0.074		18 13	15 21	0.181		17 20	16 14	0.548
**Tumor size**												
≤3cm	41	14	27	0.013^a^		24	17	0.011^a^		19	22	0.066
>3cm	26	2	24			7	19			18	8	

**Abbreviations:** MDM2, murine double minute 2; MDMX, murine double minute X; TAB1, transforming growth factor β-activated kinase 1 (TAK1)-binding protein 1; LM, lymphatic metastasis. ^a^, statistically significant.
